# Supplementary material for: Random forests, sound symbolism and Pokémon evolution
Source: PLoS One. 2023 Jan 4;18(1):e0279350. doi: 10.1371/journal.pone.0279350 (PMC9812336; doi:10.1371/journal.pone.0279350)
Supplement: Attachment — Submitted filename: ReviewPLoSONE.pdf [file pone.0279350.s001.pdf]

Review “Random Forests, Sound Symbolism and Pokémon Evolution”

Here are my comments to the authors.

1. In the Discussion section (pp.17-18), the authors discuss feature importance in the RFs they obtained across the three languages. They note that “Long vowels were important in both the Japanese and the Elicited RFs, and they skewed towards post-evolution in both cases. (p.17)”, but I would like the authors to discuss before the Experiment 2 section why the long vowel /:/ is important in the Japanese model. The study [28], as they have cited, would be helpful that found words with a long vowel to be appropriate for a post-evolved character name.

2. The authors note that “Given that the Japanese RF and the Elicited RF feature importance patterns are reasonably similar, we wanted to test whether these RFs would be able to accurately classify samples from their opposite dataset. (p.15)” I wonder how “similar” the two patterns are. If the authors want to use “similar” in this sentence, I would like them to explain that. For example, “we have five important features common to the two models.”

3. The Korean model shows that while the close back unrounded /ɯ/ was present in the post-evolution samples, the close back rounded vowel /u/ was present in the pre-evolution samples. I wonder if this difference arises from roundness of the two vowels. As the authors discuss, the Japanese model found bilabial /m/ to be suitable for the pre-evolution names (p.18). I think if the rounded vowel /u/ is assumed to have bilabiality, we understand that the presence of the bilabial (rounded) vowel /u/ in the Korean model is consistent with the presence of bilabial /m/ in the Japanese model.

4. The authors note that “Lastly, tones in the Chinese RF were important to the model. The falling tone had the highest feature importance in the Chinese RF and it skewed toward the post-evolution category. (p.17)” I wonder why the falling tone is an important feature for the post-evolution category in the Chinese model. Do you have any idea whether it is sound symbolically associated with a “big” image? I would like to the authors to explain why.

5. The authors note that “Participants were instructed not to use existing words (Japanese or otherwise) to name the Pokémon. Participants were given no further instructions (such as length limitations) regarding naming the Pokémon. (p.7) ” I wonder if all, or none, of the participants had known about Pokémon before they participated in the experiment. I would like the authors to note that they asked if the participants had played Pokémon. The experience in playing Pokémon games could yield to different results of Elicited RFs.
